# Effects of dietary vitamin E on mucosal maltase and alkaline phosphatase enzyme activities and on the amount of mucosal malonyldialdehyde in broiler chickens

**Published:** 2013

**Authors:** Seyed Hamid Farrokhifar, Ramezan Ali Jafari, Naeem Erfani Majd, Seyed Reza Fatemi Tabatabaee, Mansour Mayahi

**Affiliations:** 1 *Department of Clinical Sciences, Faculty of Veterinary Medicine, Shahid Chamran University, Ahvaz, Iran; *; 2 *Department of Basic Sciences, Faculty of Veterinary Medicine, Shahid Chamran University, Ahvaz, Iran.*

**Keywords:** Alkaline phosphatase, Broiler chicken, Malonyldialdehyde, Maltase, Vitamin E

## Abstract

The effects of dietary vitamin E levels on mucosal maltase and alkaline phosphatase (ALP) enzyme activities and on the amount of mucosal malonyldialdehyde (MDA) in broiler chickens were studied in the present study. One hundred and eighty of male day old broiler chicks (Ross 308 strain) were randomly assigned into five groups, each with three replicates and 12 chicks in each replicate. Chickens in group A were fed corn-soy- based diet, while those in groups B, C, D and E were fed the same diet with 20, 60, 180, and 540 mg kg^-1^ vitamin E supplement (d-alpha tocopherol), respectively. Six birds were randomly chosen from each group, and were euthanized on days 10, 21, 32, and 42 of age. One segment of small intestine outset was homogenized and mucosal ALP and maltase activity were measured. Moreover, mucosal lipid peroxidate amount was measured to reveal the impact of vitamin E on oxidative stress. Maltase activity was increased with the increase of vitamin E up to 60 mg kg^-1^ of diet while with further levels, it was decreased. Addition of 60 mg kg^-1^ of vitamin E to the diet significantly increased ALP enzyme activity (*p* ≤ 0.001). Addition of 540 mg kg^-1 ^of vitamin E supplement to the diet led to the minimum amount of MDA at 32 days of age. It may be concluded that supplementation of broiler's diet with 60 mg kg^-1 ^of vitamin E can increase mucosal maltase and ALP enzyme activity.

## Introduction

In recent years, the importance of gastro-intestinal system has been noticed due to its role in health and functions. Digestive enzyme activity, is one of the effective factor on performance, digestion and absorption of nutrients and finally on functions of birds.^1^

Maltase enzyme converts maltose into two molecules of glucose. Phosphatase enzymes that participate as a catalyst interact result in breaking phosphate ester molecules and separate a phosphoric acid molecule. Alkaline phosphatase in the intestinal mucosa acts as an enzyme for digestion of long chain fatty acids and cholesterol, and causes the maturation of intestinal cells.^2^^,^^3^ Appropriate pH for alkaline phosphatase (ALP) activity is about 9.0 to 9.6.^4^^,^^5^ Malonyldialdehyde (MDA) combination caused by peroxidation is produced when reactive oxygen species attack lipids and unsaturated fatty acids found in cell membranes.

Vitamin E (D-alpha tocopherol) is a fat soluble vitamin with a lot of properties necessary for natural growth and development in chickens. Vitamin E has been reported to protect cells involved in immune response, such as macro-phages, lymphocytes and plasma cells, against oxidative damage and to enhance the function and proliferation of these cells.^6^^,^^7^ Antioxidant capacity of poultry meat depends on alpha tocopherol concentration and it also depends on the level of alpha tocopherol acetate added to the diet.^8^ Generally, vitamin E supplement is added to the diet of commercial poultry in amounts of 17 mg kg^-1^ up to 48 mg kg^-1^.^9^^,^^10^ Vitamin E is hydrolyzed in the small intestine and is absorbed through the epithelium as d-alpha tocopherol. In addition to anti oxidation role in membranes, vitamin E is important in chickens as immune modulator. Increasing dose of vitamin E supplement in the diet causes higher stimulation of immune response against *E. Coli *infection,^11^ infectious bursal disease,^12^ and Newcastle disease.^13^ On the other hand, there are reports that indicate high level of vitamin E in the diet of poultry causes decrease of glutathione peroxidase activity leading to increase of free radicals in the cytosol and attenuate antioxidant defense.^14^ Additions of 150 mg kg^-1^ vitamin E to the diet of broiler chicken have led to proxidant effect and decreased antibody production.^15^

Vitamin E may affect oxidative status of the intestinal epithelium causing a change in enzyme activity and influencing absorption in gastrointestinal tract. So, the aim of this study was to determine the effects of different levels of dietary vitamin E on mucosal enzymes (maltase and ALP) activity and MDA level in broiler chickens.

## Materials and Methods

**Bird** **management. **Day-old male broiler chicks (Ross 308 strain), obtained from a commercial hatchery (Sahray-e-Jonoob broiler breeder Co., Ramhormoz, Iran), were used in the present study. This study was conducted on a commercial farm using one part of a house. This part was divided into five wired floor cages equipped with nipple drinkers, under continuous lighting system. Chickens were fed unmedicated corn-soy-based diet. The basal diet was balanced according to National Research Council.^16^ The birds were given access to water and diets ad libitum throughout the experiment. The composition of basal diet is shown in Table 1.

**Experimental design.** One hundred and eighty, day-old chicks were randomly assigned into 5 groups, each with 3 replicates and 12 chicks in each replicate with low differences in mean weight immediately after arrival to rearing hall.^17^

**Treatments.** The chicks in control group (group A) were fed on basal diet, while those in groups B, C, D and E received the same diet with 20, 60, 180, and 540 mg kg^-1^ vitamin E (D-alpha tocopherol, Aras Bazar Co., Tehran, Iran), respectively, from 1 to 42 days of age.

**Table 1:** Composition of the basal diet

**Grower ** * **(% in diet)**	**Starter ** * **(% in diet)**	**Ingredients**
**Yellow corn**	58.35	62.90
**Soybean meal**	36.60	31.00
**Vegetable oil** 1	1.00	2.20
**Dicalcium phosphate**	1.20	1.20
**Calcium carbonate**	1.20	1.20
**Oyster shell**	0.40	0.40
**Vitamin premix** 2	0.25	0.25
**Mineral premix** 3	0.25	0.25
**Salt**	0.22	0.20
**DL-Methionine**	0.25	0.20
**L-Lysine**	0.17	0.10
**Sodium bicarbonate**	0.10	0.10
**Manganese oxide**	0.005	0
**Calculated analysis**		
**Crude protein (%)**	21.50	19.20
**Metabolizable energy (kcal kg** ^-1^ **)**	2963.00	3079.00
**Methionine-Cysteine (%)**	0.83	0.74
**Lysine (%)**	1.28	1.11
**Calcium (%)**	0.92	0.90
**Available phosphorous (%)**	0.42	0.42

* The starter diet was used on days 0-21, and the grower diet on days 22-42 of study.

1 Vegetable oil contains tertiary-butylhydroquinone (TBHQ), 120 ppm as antioxidant.

2 Vitamin premix provides the following ingredients per kg of diet: Vitamin A, 9000 IU; Vitamin D_3_, 2000 IU; Vitamin E (alpha tocopherol acetate), 18.00 IU; Vitamin K_3_, 2.00 mg; Thiamine, 1.80 mg; Riboflavin, 6.60 mg; Calcium pantothenate, 10.00 mg; Niacin, 30.00 mg; Vitamin B_6_, 3.00 mg; folic acid, 1.00 mg; vitamin B_12_, 0.01 mg; biotin, 0.10 mg; choline chloride, 50.00 mg.

3 Mineral premix provides the following ingredients per kg of diet: iodine, 1 mg; selenium, 0.20 mg; iron, 50.00 mg, copper, 10.00 mg; zinc, 100 mg; manganese, 100 mg.

**Sample collection.** On days 10, 21, 32 and 42 of rearing period, after 3 hr of fasting,^18^ two chicks from each replicate (6 birds per treatment) were randomly sacrificed by cervical dislocation. Immediately after euthanasia, a 10 cm piece of small intestine (from duodenal loop) was cut open lengthwise, rinsed carefully with cold phosphate buffered saline (0.1 M, pH 7), blotted dry, enveloped in vacuum pack and stored at -80 ˚C until enzyme analysis. To determine the intestinal mucosal enzymes activity, after thawing, all of vacuum packs were opened and then the mucosa were gently scraped and 50 mg of the mucosa was homogenized using an ultrasonic homogenizer (Bandelin Electronic, Berlin, Germany) in 3.0 mL 0.1 M phosphate buffered solution, pH 7. Alkaline phosphatase activity was measured by commercial kit (Parsazmun Co., Karaj, Iran). This kit is designed for measuring up to 0.25 optical absorptive variations in one minute. Alkaline phosphatase acts on colorless substrate (Para nitro-phenyl phosphate) and converts it to phosphate and para nitrophenol which is yellow in alkaline pH. Optical absorption is recorded after 1 min according to kit's instruction. Severity of color density depends on the ALP activity. The length of time for the assay of Maltase was specified by Dahlqvist with modifications.^19^^,^^20^ The homogenized mucosa was added to the substrate and the amounts of produced glucose were determined by commercial kit (Parsazmun Co., Karaj, Iran). Briefly peroxidase acts on the hydrogen peroxide (released from glucose by glucose oxidase), 4-amino-antipyrine and phenol. Produced quinoneimine is photometric measurable and depends on the amount of glucose. Finally, for detection of enzyme activity it was needed to measure total protein in homogenized mucosa tissue using commercial kits assayed by pyrogallol method to express the activity of each enzyme per gram of protein.^21^ To determine the effect of vitamin E on oxidative stress status, total amount of lipid peroxidation in mucosal tissue was measured by thiobarbituric acid method.^22^

**Statistical** **analysis**. The results of the experiment

were analyzed by multivariate analysis of variance by using the linear model of SPSS software (SPSS 16.0 Student version for windows, Surrey, UK). Differences in variables were attained using Tukey's HSD post hoc test and *p *≤ 0.05 was considered statistically significant. All averages as well as the mean ± SD were presented. 

## Results

**Effects of treatments on mucosal maltase activity. **Both group and age had significant effects on maltase activity (*p *< 0.001) and their interaction was also significant (*p *< 0.01). Increasing the level of vitamin E up to 60 mg kg^-1^ and 180 mg kg^-1^ (*p *≤ 0.001) significantly increased total mean maltase activity. However, 540 mg kg^-1^ returned it to the control group level. There was a significant difference between control with 60 mg kg^-1 ^(*p *< 0.001) and 180 mg kg^-1 ^groups (*p *< 0.01), (Table 2). Minimum activity of maltase was observed on days 21 and 32 of age (*p *< 0.001) (Table 2). 

**Effects of treatments **on** mucosal ALP activity. **Both group and age had significant effects on ALP activity and their interaction was also significant (*p *< 0.001). Addition of 60 mg kg^-1 ^of vitamin E to the diet significantly increased total mean ALP enzyme activity compared to the control group (*p *≤ 0.001). The minimum ALP activities were seen on 42 days old chicks (Table 2).

**Effects of treatments on MDA level. **Both group and age had significant effects on MDA level (*p *< 0.001) and their interaction was also significant (*p *< 0.001). Increasing the level of vitamin E reduced MDA level (*p *< 0.001) (Table 2). The higher level of MDA was observed on day 21 of age which was significantly different from those of 32 and 42 days of age (*p *< 0.001). 

**Table 2:** The effects of different dietary levels of vitamin E on mucosal maltase, ALP, and MDA in broiler chickens (Mean ± SD).

**Parameters Vitamin E (mg kg** ^-1^ **) 10 days old**			** 21 days old**	** 32 days old**	**42 days old**
**Maltase** ** (** **IU** **) ** **per** **gram of protein**	01	298 ± 49.00	127 ± 27.00	161 ± 30.00	329 ± 146
	20	353 ± 64.00^ns^	195 ± 53.00*	163 ± 40.00^ns^	404 ± 90.00^ns^
	60	446 ± 59.00*	137 ± 33.00^ns^	300 ± 64.00***	535 ± 49.00*
	180	459 ± 117**	132 ± 18.00^ns^	250 ± 52.00^ns^	406 ± 93.00^ ns^
	540	398 ± 49.00^ ns^	129 ± 35.00^ns^	151 ± 70.00^ns^	371 ± 102^ ns^
**Alkaline phosphatase** ** (IU) per gram of protein**	0	7903 ± 5539	12260 ± 2420	15079 ± 4054	10626 ± 2060
	20	18604 ± 1368^ns^	15340 ± 3354^ns^	11036 ± 2375^ns^	528 ± 1679^ns^
	60	18688 ± 3775^ns^	17043 ± 2981*	24202 ± 6805**	11988 ± 2948^ns^
	180	19724 ± 6087^ns^	15033 ± 2325^ ns^	13122 ± 1166^ns^	164 ± 1517^ns^
	540	17674 ± 4414^ns^	10166 ± 2183^ ns^	9690 ± 2264^ ns^	6587± 1617*
**Malonyldialdehyde (** **µmol) per gram of protein**	0	1.87 ± 0.79	2.13 ± 0.54	1.45 ± 0.67	2.22 ± 0.57
	20	1.96 ± 0.59^ ns^	1.65 ± 0.47^ ns^	0.89 ± 0.21^ ns^	1.34 ± 0.58**
	60	1.44 ± 0.13^ ns^	1.80 ± 0.46^ ns^	1.89 ± 0.36^ ns^	0.39 ± 0.17***
	180	0.78 ± 0.28**	1.00 ± 0.27***	0.93 ± 0.30^ ns^	0.76 ± 0.17***
	540	0.43 ± 0.12***	0.98 ± 0.19***	0.25 ± 0.03***	0.76 ± 0.11***

Levels of significance compared to the control group in each column: ^ns^ non-significant (*p *> 0.05),

* (*p *≤ 0.05),

** (*p *≤ 0.01),

*** (*p *≤ 0.001).

1 Control group was fed with un-medicated conventional corn-soy-based diet, as in Table 1.

## Discussion

According to Table 2, addition of different levels of vitamin E supplement to the diet of broilers at various ages caused a variety of influences on the activity of maltase and ALP enzymes and MDA level.

Addition of vitamin E supplement to the diet of birds at 10 (60 and 180 mg kg^-1^) and 21 day old (20 mg kg^-1^) increased maltase enzyme activity (*p *< 0.05, *p *< 0.01 and *p *< 0.05) and, also, addition of vitamin E supplement (60 mg kg^-1^) to the diet of birds on 32 (*p *≤ 0.001) and 42 days of age (*p *< 0.05) increased maltase enzyme activity. Addition of vitamin E supplement (60 mg kg^-1^) to the diet of birds on 42 days of age led to the greatest level of maltase enzyme activity compared to that of the control group. The obtained results indicated that the addition of vitamin E supplement (60 mg kg^-1^) to feed had a significant effect on the small intestine maltase activity, such as 10 (*p *< 0.05), 32 (*p *≤ 0.001), and 42 days of age (*p *< 0.05) at the small intestine mucosa (Table2).

Addition of 60 mg kg^-1 ^of vitamin E to the diet significantly increased total mean ALP enzyme activity compared to that of the control group (*p *≤ 0.001), (Table2).

These findings are possibly as a result of the effect of vitamin E supplement on the intestinal microflora such as higher stimulation of immune response against *E. Coli *infection. This type of bacterium may damage the villi of intestinal mucosa and inhibit the secretion of digestive enzymes.^23^^,^^24^ Thus, according to Erf *et al*. and McIlory studies these findings, to some extent, confirm the amount of 17 up to 48 mg kg^-1^ of vitamin E supplement in the diet of commercial poultry.^9^^,^^10^

Malonyldialdehyde level, as a general indicator of lipid peroxidation, is increased with increasing oxidative stress. The minimum amount of MDA was observed at 32 days of age. However, in this study, although 540 mg kg^-1^ of vitamin E was added to the diet, minimum amount of MDA was seen in all ages of sampling and it was the lowest at 32 days of age among them.

Thus, the greatest effect of vitamin E to prevent oxidation up to 32 days old was observed when 540 mg kg^-1 ^of vitamin E supplement was added to the diet. But, it is interesting to note that addition of 180 mg kg^-1 ^of vitamin E to the diet reduced the amount of MDA as did the addition of 540 mg kg^-1^ of vitamin E in the diet. Thus, the effects of addition of 180 mg kg^-1^ of vitamin E to the diet are almost as effective as those of 540 mg kg^-1^ (Table2).

Several reports suggest the reduction of alpha tocopherol in the liver and serum of young chickens and poults, in the first two weeks of life.^25^^,^^26^ Thus, a high level of vitamin E on the first 10 days of rearing is necessary. As was confirmed in this study, high doses of vitamin E are needed to increase the enzyme activity of mucosal maltase and to reduce the amount of MDA (as lipid peroxidation index) on the 10 days of rearing, however, increasing of ALP enzyme activity compared to the control was not significant (*p *> 0.05), (Table 2). According to Leshchinsky and Klasing studies, it is reported that the addition of high amounts of vitamin E in poultry diets reduced glutathione peroxidase activity in the cytosol of the cell, which resulted in the increase of free radicals and weakening of antioxidant defenses.^14^ Also, Friedman *et al*. found that with the addition of 150 mg kg^-1 ^of vitamin E to the diet of broiler chickens, antibodies production was decreased and peroxidation effects was observed in this level.^15^ Thus, increasing the amount of MDA in the D (180 mg kg^-1^ vitamin E) and E (540 mg kg^-1^ vitamin E) groups that received more than 60 mg kg^-1^ of vitamin E in the diet can be considered as the onset of peroxidation effect of vitamin E on 42 days of age (Table2).

Our results suggested that high doses of vitamin E are needed to increase enzyme activity of mucosal maltase and to reduce the amount of MDA (as lipid peroxidation index) on the first 10 days of rearing. This study showed that addition of more than 60 mg kg^-1^ of Vitamin E supplement to the diet can be considered as the onset of peroxidation effect of vitamin E at older ages. The treatments under investigation (60 mg kg^-1^) could be used to increase total mean ALP and maltase activity. Also, these levels of vitamin E could reduce the amount of MDA on 42 days of age (as lipid peroxidation index). 
